# Venous dilation effect of hot towel (moist and dry heat) versus hot pack for peripheral intravenous catheterization: a quasi-experimental study

**DOI:** 10.1186/s40101-023-00340-5

**Published:** 2023-10-19

**Authors:** Kae Yasuda, Inaho Shishido, Michito Murayama, Sanae Kaga, Rika Yano

**Affiliations:** 1 Graduate School of Health Sciences, Hokkaido University, N12, W5, Kita-Ku, Sapporo, Hokkaido 060-0812 Japan; 2 Faculty of Health Sciences, Hokkaido University, N12, W5, Kita-Ku, Sapporo, Hokkaido 060-0812 Japan

**Keywords:** Heating, Hot towel, Hot pack, Venous dilation, Vein, Skin temperature, Peripheral intravenous catheterization

## Abstract

**Background:**

Heat application before peripheral intravenous catheterization is recommended for venous dilation. Hot pack application enlarges the venous diameter in healthy adults; however, hot towels (moist and dry heat) are used often in some medical cases. However, it is unclear whether hot towel application promotes venous dilation better than hot pack application. This study compared the venous dilation effect of using a hot towel (moist and dry heat) to a hot pack before applying the tourniquet at an access site for peripheral intravenous catheterization.

**Methods:**

Eighty-eight healthy females aged 18–29 years were recruited for this quasi-experimental study. They underwent three types of heat applications (hot pack, moist hot towel, and dry hot towel [moist hot towel wrapped in a dry plastic bag], all of which were warmed to 40 ± 2 °C and performed for 7 min) to their forearm and tourniquet application for 30 s after each heating. Venous diameter and depth were measured using ultrasonography, and venous palpability and visibility (venous assessment score) was observed as venous dilatation effects. In addition, the skin temperature, stratum corneum hydration, and subjective evaluation of the warmth were measured.

**Results:**

There were no significant differences in venous diameter and assessment scores after intervention between the dry hot towel and the hot pack groups, and the effect size was negligible (Cohen’s d < 0.20). However, these measurements were significantly lower for the moist hot towel than for the other two heat applications (*P* < .001). Although there was no significant difference in skin temperature and warmth rating score between the dry hot towel and the hot pack, these were significantly lower for the moist hot towel than for the other two heat applications (*P* < .001). The amount of change in stratum corneum hydration of the dry hot towel was not significantly different from that of the hot pack; however, that of the moist hot towel was significantly larger than that of the other two heat applications (*P* < . 001.)

**Conclusions:**

A method in which a towel warmed in hot water is wrapped in a dry barrier may be an alternative to a hot pack.

**Trial registration:**

This study was registered with University Hospital Medical Information Network in Japan (Registration No.: UMIN000048308. Registered on July 7, 2022).

**Supplementary Information:**

The online version contains supplementary material available at 10.1186/s40101-023-00340-5.

## Background

According to the National Home Infusion Association (2023), the practice of infusion therapy has shifted to in-home care settings in recent years because of cost-effectiveness and personal lifestyle considerations [1]. The home infusion therapy market was valued at US$31 billion in 2021 and is expected to increase to US$61.7 billion by 2030 [2]. Insertion of a peripheral intravenous catheter (PIVC) is a basic medical procedure that is less invasive than central venous access. However, complications such as extravasation and occlusion occur in 75% of home care patients who undergo PIVC insertion, and half of them experience multiple insertions because of failure at the first attempt [3]. Most medical professionals select the insertion site for a PIVC by observing and palpating the vein. Thin, poorly palpable, or poorly visible veins make PIVC insertion difficult despite the use of a tourniquet [4]. Therefore, it is important to dilate the vein sufficiently to safely insert the PIVC [5].

Before using a tourniquet at the access site for PIVC insertion, heat application is recommended as a venous dilation procedure [6]. When thermal energy is transferred to the skin, venodilators such as nitric oxide are released, and the blood flow in the cutaneous veins increases [7]. In terms of safety and venous dilation effects, the surface temperature of heated items applied to the skin is recommended to be 40 ± 2 °C [5]. Hot packs have the highest utilization rate for heat applications in hospitals (65%), but hot towels are also used often not only in America but also in Japan [8]. Hot towel applications include two methods: direct application of a towel moistened with hot water (moist heat) and wrapping it in a dry barrier and applying it (dry heat) [8].

Previous studies on the venous dilation effects using a hot pack (gel sealed in dry plastic) in the forearm cutaneous veins of healthy adults revealed the following: 1) in a randomized controlled trial, using a tourniquet after 15 min of heat application significantly enhanced venous cross-sectional area dilation than using tourniquet alone [9], 2) there was no significant difference in the venous dilation effect of application times between 5 and 15 min [10], and 3) the venous dilation effect persisted for at least five minutes after the removal of heat in the 15 min application [11]. For heat application using hot towels, the effectiveness of the moist and dry heat methods was compared in a randomized controlled trial involving 136 hematology outpatients [12]. The results showed that the success rate of the first PIVC insertion was significantly higher under dry heat than under moist heat. However, the venous palpability and visibility after heating were not significantly different. A PIVC insertion is associated with the promotion of venous dilatation and improvement venous palpability and visibility [4, 13]. Therefore, it is unclear whether the dilated veins increased the success rate of PIVC insertion in the context of dry heat. Because no study involved a control group that underwent the standard procedure (only tourniquet application), it is also unclear how many veins dilate when using the hot towel.

Heat transfer to the skin seems to be affected not only by temperature but also by the material of the item and moist and dry heat stimulation. However, it is unclear how extensively veins dilate when using hot towels and whether hot packs or hot towels are more effective heat application methods for venous dilation. As these methods are mixed in medical practice, it is necessary to show evidence of a more effective method that leads to secure PIVC insertion.

This study compared the venous dilation effect of using a hot towel (moist and dry heat) to a hot pack before applying the tourniquet at an access site for PIVC insertion. This study provides basic evidence that heat application methods should be selected for PIVC insertion to promote greater venous dilatation. In addition, if hot towels provide the same venous dilation effect as hot packs, using hot towels instead of hot packs may be recommended in resource-limited situations, such as home nursing and disaster situations.

## Methods

### Study design

This quasi-experimental study followed the Transparent Reporting of Evaluations with Nonrandomized Designs guidelines (see Additional file 1). After each of the three heat applications (hot pack: using a hot pack, moist hot towel: using a face towel warmed with hot water, dry hot towel: using a moist hot towel wrapped in a plastic bag), a tourniquet was applied to each participant’s forearm. Allocations of heat applications were assigned using computer-generated random numbers assigned by an independent study associate. Experiments were performed on three consecutive days whenever possible. The washout period for the heat applications was 24 h. Each heat application was performed at the same time between 10:00 a.m. and 4:00 p.m. to minimize its influence on the autonomic nervous system [14]. It was impossible to blind the participants to the intervention because it involved thermal intervention. The evaluator was not blinded because they were the same person as the interventionist.

### Participants

Ninety healthy female students aged 18–29 years who visited a Japanese national university between July 2022 and November 2022 were recruited as a convenience sample. This study aimed to control for differences in venous degeneration and reactivity caused by aging and hormonal balance between sexes [15, 16]. Females have smaller vessel diameters than males [17, 18] and it is more challenging to insert PIVCs in females [19]. The exclusion criteria were as follows: 1) a history of cardiovascular disease; and 2) receiving any treatment on the forearm skin because of severe skin diseases, injuries, or eczema. Participants were instructed to refrain from alcohol consumption within eight hours and from eating, consuming caffeine or stimulants, and engaging in strenuous exercise or showering within an hour before the experiment initiation.

### Sample size

Based on the difference in venous diameter between heat applications in an unpublished pilot study, 88 participants were required in this study [dry hot towel vs. hot pack: two-sided significance of 0.05, power of 80%, and effect size of 0.50, Student’s t-test]. We enrolled 90 participants after considering the dropout rate. The sample size was determined using the G Power software version 3.1.9 (G*Power, Heinrich Heine University, Düsseldorf, Germany).

### Target vein

The cephalic, median, or basilic veins of the non-dominant arm were selected, which were ≤ 30 mm distal from the antecubital fossa, ≤ 120 mm proximal to the radial styloid, and as large as possible. In addition, the vein was straight ≥ 2.5 mm, lying ≤ 10 mm deep, and with a diameter ≥ 0.9 mm [20–24]. This criterion assumes a 22-gauge thick and 25 mm long catheter insertion (BD Insyte Autoguard™ BC Shielded IV Catheter with Blood Control Technology, Nippon BD Co., Tokyo, Japan). The measurement sites were marked with surgical tape and photographed whenever possible to ensure that the same position could be measured.

### Measurement environment

This study was conducted in a measurement room at a university in Japan. The measurement environment was a standard hospital room. The temperature was 22–24 °C, the humidity was 45–60%, and illuminance was set to 185–215 lx in this room based on Japanese industrial standards [25].

### Procedure

The intervention methods were based on previous studies of heat application before PIVC insertion [9, 12]. One researcher conducted all the interventions in a unified manner.

#### Heat application -1: Hot pack

1) A hot pack (gel sealed in plastic: 3M^TM^, Cold/Hot Pack, 10 cm × 25 cm, 290 g, 3M Health Care, Tokyo, Japan) was heated at 40 ± 2°C in a thermostatic chamber filled with hot water (46℃) and patted with a dry towel. 2) It was applied to the participant’s forearm for seven minutes. 3) After discontinuing heating, the participants were instructed to rest for 4 min and 30 s. 4) A tourniquet (TTQ‐100‐1, TAIYO Instruments INC, Osaka, Japan) was placed 10 cm proximal to the measurement site [26] and a constant pressure of approximately 75 mmHg was applied for 30 s [27].

#### Heat application -2: Moist hot towel

1) A face towel (48 × 66 cm, 81 g, dry), folded into a size of 11 × 24 cm was soaked in a thermostatic chamber filled with hot water (46℃), and wrung out to 290 ± 5 g (the surface temperature of the towel was 40 ± 2°C). 2) It was applied to the participants’ forearm for seven minutes. The following interventions were the same as in 3) and 4) for heat application: 1.

#### Heat application -3: Dry hot towel

1) The moist hot towel was wrapped in a dry plastic bag (size, 25 × 35 cm; thickness, 0.02mm, hereafter referred to as the “Dry-hot towel”). The surface temperature of the dry hot towel was also 40 ± 2°C. 2) It was applied to the participants’ forearm for seven minutes. The following interventions were the same as in 3) and 4) for heat application: 1.

The heating method applied to each item in this study was validated to maintain a consistent temperature of 40 ± 2°C using thermography (R300, Nippon Avionics Co., Ltd., Yokohama, Japan). This validation was accomplished through a preliminary process conducted 20 times for each item (Mean [SD], Hot pack: 41.4 [0.4] °C, Dry hot towel: 41.1 [0.6] °C, Moist hot towel: 41.0 [0.6] °C). Therefore, the surface temperatures of the three items were comparable. To ensure temperature stability, no measurements were taken during each intervention, mitigating any potential decrease in surface temperature after preparation of the items. Applying 40 ± 2°C to the skin increases blood flow in the cutaneous veins [7]; this temperature is also lower than 44°C, which can lead to skin burns [28]. The resting time between heat application and tourniquet application was considered the time required to remove the heat applied during the actual PIVC insertion [29–31]. The participants were instructed to refrain from forearm movements, such as clenching a fist, which affects venous dilation.

### Data collection (Fig. 1)

**Fig. 1:**
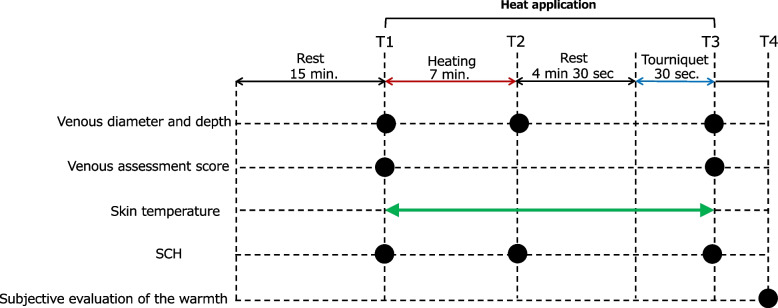
Protocol of the data collection. Measurements performed at each time point are shown in parentheses "●". Participants’ characteristics were measured before heating on the first day. SCH, stratum corneum hydration; T1, baseline; T2, after heating; T3, after applying the tourniquet; T4, at the end of each measurement day

#### Venous diameter and depth

**Fig. 2:**
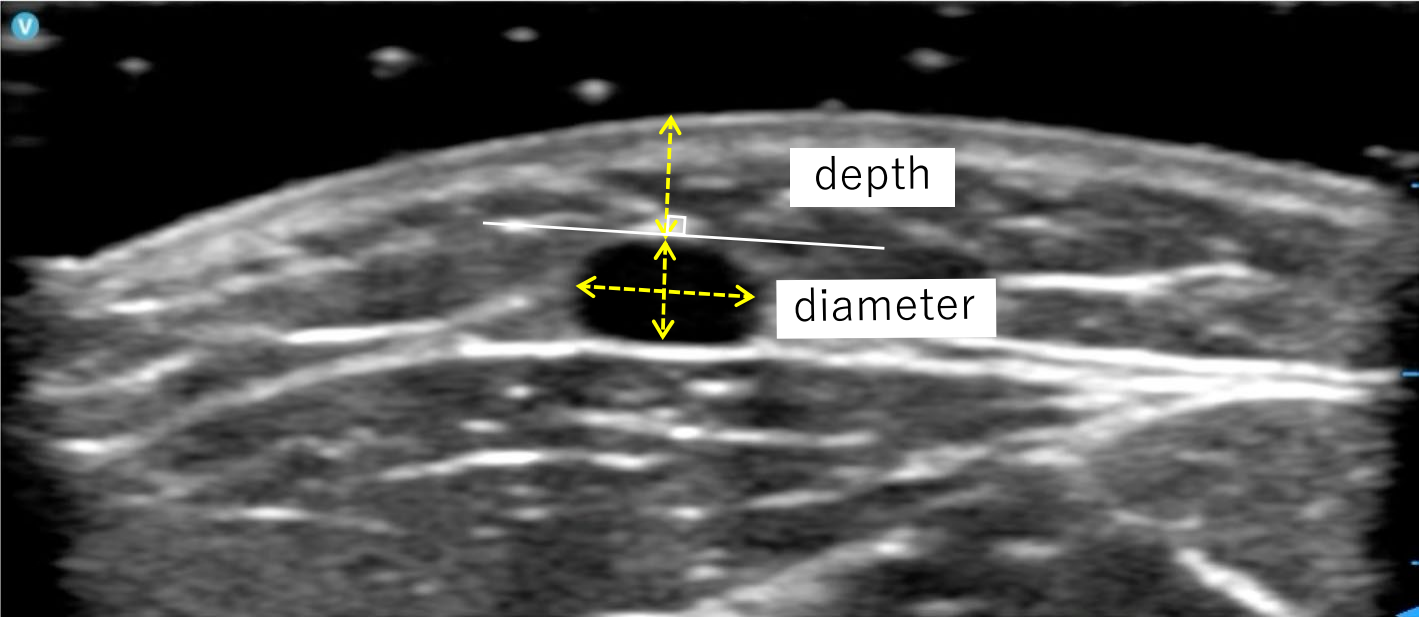
Measuring the venous diameter and the venous depth. The venous diameter (mm) was calculated as the average of the shortest diameter (from the upper end to the lower end of the vein) and the longest diameter (perpendicular to the shortest diameter) (= [shortest diameter + longest diameter] / 2). Venous depth (mm) was measured as the vertical distance between the skin surface and the upper end of the vein

The primary outcome measure was the venous diameter, a success factor for PIVC insertion [4]. Venous depth was measured as a secondary outcome. These were measured using portable ultrasonography (Vscan Air, 11-3L probe, GE Healthcare, Little Chalfont, UK) before the intervention (baseline, T1), after the removal of heat (after heating, T2), and at 30 s of tourniquet application (after applying the tourniquet, T3). The venous diameter (mm) was calculated as the average of the shortest diameter (from the upper end to the lower end of the vein) and the longest diameter (perpendicular to the shortest diameter) (= [shortest diameter + longest diameter] / 2). Venous depth (mm) was measured as the vertical distance between the skin surface and the upper end of the vein (Fig. 2). One researcher with experience in measuring cutaneous vein size in previous studies [24, 32] performed the measurements in this study under the guidance of a study associate who was a registered medical sonographer in cardiology and gastroenterology with more than eight years of experience. The high reliability of the measurements was confirmed before this study (ICC [2.1]; venous diameter, 0.975; venous depth, 0.929). Portable ultrasonography was performed after confirming the reliability and validity of the measurements using previously used stationary ultrasonography (Aplio XG SSA-790A, PLT-1204AT probe, Canon Medical Systems, Tochigi, Japan). Before the measurement, the gel was warmed to approximately the temperature of the participant's skin surface using a gel warmer.

#### Venous assessment score

The venous assessment score, which evaluates the palpability and visibility of veins on a 5-point scale [33], was used at baseline (T1) and after applying the tourniquet (T3). This score was evaluated in a previous study that compared moist and dry hot towels [12]. The evaluation degrees were as follows:1) veins are neither visible nor palpable; 2) veins are visible but not palpable; 3) veins are barely visible and palpable; 4) veins are visible and palpable; and 5) veins are clearly visible and easily palpable. One researcher who was a licensed nurse with at least three years of clinical experience standardized the evaluation. The researcher practiced for several days following prior instructions from an expert nurse and midwife with at least five years of clinical experience [34]. Given the remarkable agreement rate of 0.998 (weighted kappa coefficient) between the assessments by the researcher and their collaborator on the 26 data points, we concluded that there was no problem with the researcher's assessment skill.

#### Skin temperature on the heat application site

A skin surface thermometer (Hardware N543, NIKKISO-THERM CO. LTD., Tokyo, Japan) was used at the intervention site to measure the changes in skin temperature at the heat application site over time (T1**–**T3). The measurement probe was placed at a location that did not overlap the venous measurement site within the range of the thermal item.

#### Stratum corneum hydration

Stratum corneum hydration (SCH) was performed using a Corneometer® CM825 (Courage + Khazaka, Cologne, Germany) at baseline (T1), after heating (T2), and after applying the tourniquet (T3) to identify changes in water content in the stratum corneum of the forearms. The measurements were repeated five times, and the mean of the three values obtained was calculated, excluding the maximum and minimum values.

#### Subjective evaluation of the warmth

The participants were instructed to recall and evaluate the degree of forearm warmth experienced during each heat application at the beginning, just before the heat removal, and after the applying the tourniquet. These were evaluated at the end of each measurement day (T4). The evaluation degrees of the warmth were as follows: 0 = feeling cold, 1 = feeling cold slightly, 2 = feeling nothing, 3 = feeling a little warmth, and 4 = feeling warmth.

#### Participants’ characteristics

The participants’ characteristics were measured on the first day. The participants were asked to provide information about their age, height, and experience with peripheral venipuncture (blood sampling or PIVC insertion) failure using a 4-point scale: never, rarely, sometimes, and often [35]. Body weight, body mass index (BMI), body fat percentage, and muscle mass were measured using a body composition analyzer (RD-917; TANITA, Tokyo, Japan). Blood pressure and pulse were measured using an electronic sphygmomanometer (ES-H56; TERUMO, Tokyo, Japan), and body temperature was measured using an electronic thermometer (MC-681; OMRON, Kyoto, Japan). The change in venous diameter before and after tourniquet application was measured before heat application (tourniquet only). The washout period between the use of a tourniquet alone and heat application was 15 min [24].

### Data analysis

For descriptive statistics, the mean (standard deviation [SD]; 95% confidence interval [CI]) and frequency (%) of categorical variables were used. Least square mean (LSM) was calculated using the mixed model. In terms of SCH, changes in heating (T2 – T1) and changes after removed heating (T3 – T2) were calculated. The skin temperature was calculated every 30 s from T1 to T3. All data were analyzed using the JMP® Pro software, ver.16.1 (SAS Institute Inc., Cary, NC, USA). The significance level was set at *P* ≤ 0.05. For comparisons between heat applications, Bonferroni correction was performed, and *p*-values were considered significant at α = 0.05 / 3 = 0.0167.

First, a one-way repeated ANOVA and Dunnett's test (tourniquet only = control, adjusted for baseline values) using a mixed linear model were used to confirm the dilating effect of each heat application compared with tourniquet only on changes in the venous diameter and venous depth (= after tourniquet ‒ baseline).

The following comparisons between the heat applications were performed: venous diameter, venous depth, skin temperature, and subjective evaluation of the warmth were analyzed using a two-factor repeated measures analysis of variance (ANOVA) with a mixed-linear model. In this model, the dependent variables were the venous diameter, depth, skin temperature, and subjective evaluation of the warmth. The fixed effects, conditions, times, and interactions were determined. The participants were statistically assigned to random effects. One-way repeated ANOVA and Bonferroni correction were used to compare interventions at each time point. Comparisons between time points within groups were performed using one-way repeated ANOVA and Dunnett's test (baseline = control). The amount of change in SCH was analyzed using one-way repeated ANOVA and Bonferroni correction. In this model, the change in the amount of SCH is the dependent variable. The effect size (Cohen’s d) of these group comparisons of venous diameter and depth. Cohen’s d was interpreted as negligible (< 0.2), small (0.2‒0.4), medium (0.5‒0.7), and large (≥ 0.8) [36]. Venous assessment scores were compared between heat applications within time points and between time points within heat applications (T1 vs. T3) using the Freeman–Halton extension of Fisher’s exact test and Bonferroni correction.

## Results

### Participant characteristics

**Fig. 3:**
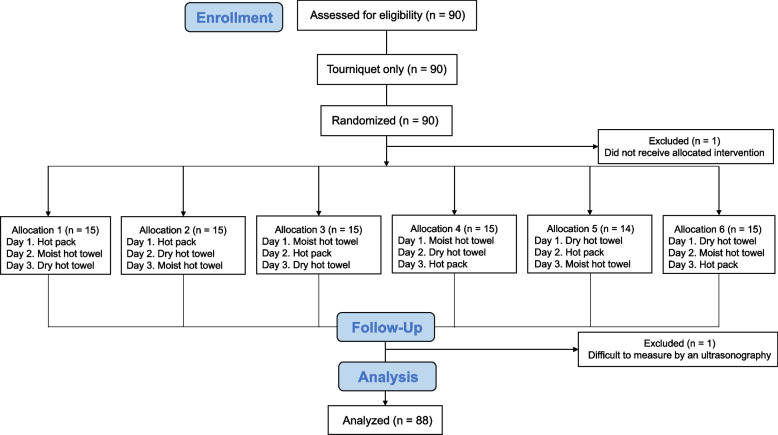
Diagram of the study flow

This study was able to complete the measurements in 89 of the 90 participants. One participant was excluded because her veins did not meet the measurement criteria and 88 participants were included in the analysis (Fig. 3). All participants had an average BMI of 20.2 (2.4) kg/m^2^ and a body fat percentage of 25.3 (4.9) %, which is the standard for Japanese female in their twenties (Table 1). Venous sizes before and after the tourniquet are shown in Table 1. No adverse events such as burns occurred with the use of these heat applications.

**Table 1:** Participant's characteristics (*n* = 88)

Characteristic	n	(%)	Mean	(SD)
Dominant forearm				
Right	81	(92.0)	-	
Left	7	(8.0)	-	
Age (years)	-		21.7	(2.3)
BMI (kg/m^2^)	-		20.2	(2.4)
Body fat percentage (%)	-		25.3	(4.9)
Body muscle mass (%)	-		35.7	(4.0)
Body temperature (°C)	-		36.6	(0.2)
Forearm area (cm^2^)	-		170.5	(18.3)
Pulse rate (beats/min)	-		67.4	(11.5)
Systolic BP (mmHg)	-		100.4	(7.5)
Diastolic BP (mmHg)	-		75.7	(18.5)
Venous diameter (mm)				
Before Tourniquet only	-		3.16	(0.77)
After Tourniquet only	-		3.51	(0.84)
Venous depth (mm)				
Before tourniquet only	-		4.12	(1.55)
After tourniquet only	-		3.82	(1.45)
Failure of venipuncture				
Never	60	(68.2)		
Rarely	7	(8.0)		
Sometimes	7	(8.0)		
Often	14	(15.9)		

*BMI* body mass index, *BP* blood pressure, *SD* standard deviation

### Comparison of the venous dilation effects

In terms of venous diameter, the amount of change between baseline (T1) and after tourniquet (T3) application was found to be significantly greater for both hot pack (LSM: 0.59 [0.50–0.68] mm) and the dry hot towel (0.50 [0.42–0.59] mm) was significantly larger (*P* < .001, *P* = .016) than that observed with the tourniquet only (0.35 [0.26–0.43] mm). Conversely, the moist towel exhibited a significantly smaller alteration (0.09 [0.00–0.18] mm) compared to tourniquet only (*P* < .001). Upon comparing the various heat application methods, notable main effects were identified for heat application (F [2, 694.0] = 25.11, *P* < .001), time (F [2, 694.0] = 78.97, *P* < .001), and interaction (F [4, 694.0] = 12.50, *P* < .001, Table 2). No significant difference was observed between using the hot pack and the dry hot towel, and the effect size was negligible (T2: mean [95% CI] mm of difference:0.06 [-0.19–0.07], d = 0.12, *P* = .448, T3: 0.12 [-0.25–0.02], d = 0.17, *P* = .120). However, the moist hot towel application was significantly smaller than the hot pack at T2 and T3 (T2: *P* = . 006, T3: *P* < .001). The moist hot towel was also significantly smaller than the dry hot towel at T3 (*P* < .001).

**Table 2:** The venous diameter of hot towel (moist and dry heat) versus hot pack (*n* = 88)

Venous diameter (mm)		Time						Main effect				Interaction Condition × Time	
		**T1**^**b**^		**T2**		**T3**		**Condition**		**Time**			
		**Baseline**		**After heating**		**After tourniquet**		***F(df)***	***P***^***a***^	***F(df)***	***P***^***a***^	***F(df)***	***P***^***a***^
Hot pack	**Mean (SD)**	3.15	(0.76)	3.39	(0.77) *	3.75	(0.85) *	25.11 (2,694.0)	** < .001**	78.97 (2,694.0)	** < .001**	12.50 (4,694.0)	** < .001**
Dry hot towel		3.13	(0.81)	3.34	(0.84) *	3.63	(0.84) *						
Moist hot towel		3.17	(0.76)	3.23	(0.74)	3.26	(0.86)						
Hot pack vs. Dry hot towel	**MD [95%CI]**	-0.02	[-0.09–0.05]	-0.06	[-0.19–0.07]	-0.12	[-0.25–0.02]						
	***t*, d**	-0.77	,0.08	-1.09	,0.12	-1.84	,0.17						
	***P*** ^**c**^	.658		.448		.120							
Hot pack vs. Moist hot towel	**MD [95%CI]**	0.02	[-0.05–0.09]	-0.17	[-0.30–0.05]	-0.49	[-0.63–0.35]						
	***t*, d**	0.53	,0.06	-3.01	,0.32	-7.89	,0.84						
	***P*** ^**c**^	.816		**.006**		** < .001**							
Dry hot towel vs. Moist hot towel	**MD [95%CI]**	-0.04	[-0.10–0.02]	0.11	[-0.00–0.23]	0.38	[0.25–0.51]						
	***t*, d**	-1.32	,0.04	1.93	,0.14	5.90	,0.46						
	***P*** ^**c**^	.188		.056		** < .001**							

*C*I conference interval, *df* degree of freedom, *MD* mean difference, *SD* standard deviation

^a^A mixed-linear model for two-way repeated-measures ANOVA

^b^One-way repeated-measures ANOVA and Dunnett's test (T1 = control; * P < .01)

^c^One-way repeated-measures ANOVA and Bonferroni correction (*P* < .0167)

Regarding venous depth, there was also no significant difference between the tourniquet only and each heat application at T3 (LSM [95%CI]; tourniquet only: -0.30 [-0.19 – -0.40] mm, hot pack: -0.35 [-0.24 – -0.45] mm, *P* = .955; dry hot towel: -0.27 [-0.16 – -0.37] mm, *P* = .828; moist hot towel: -0.26 [-0.16 – -0.37] mm, *P* = .934). When a comparison was made among the different heat applications, a significant main effect was observed solely for time (F [2,693.0] = 35.50, *P* < .001, Table 3). All three heat treatments were significantly shallower from T1 to T3 (*P* < .01, Table 3).

**Table 3:** The venous depth of hot towel (moist and dry heat) versus hot pack (*n* = 88)

Venous depth (mm)		Time						Main effect				Interaction Condition × Time	
		**T1**^**b**^		**T2**		**T3**		**Condition**		**Time**			
		**Baseline**		**After** **heating**		**After tourniquet**		***F(df)***	***P***^***a***^	***F(df)***	***P***^***a***^	***F(df)***	***P***^***a***^
Hot pack	**Mean (SD)**	4.19	(1.58)	4.04	(1.51)	3.84	(1.48) *	1.08 (2,693.0)	.340	35.50 (2,693.0)	** < .001**	0.49 (4,693.0)	.741
Dry hot towel		4.19	(1.55)	4.15	(1.64)	3.92	(1.52) *						
Moist hot towel		4.18	(1.47)	4.04	(1.52)	3.91	(1.44) *						
Hot pack vs. Dry hot towel	**MD [95%CI]**	0.00	[-0.14–0.14]	0.08	[-0.06–0.21]	0.08	[-0.07–0.23]						
	***t*, d**	0.02	,0.00	1.26	,0.13	1.19	,0.13						
	***P*** ^**c**^	.999		.989		.389							
Hot pack vs. Moist hot towel	**MD [95%CI]**	-0.01	[-0.14–0.13]	0.01	[-0.13–0.15]	0.08	[-0.08–0.23]						
	***t*, d**	0.02	,0.01	0.12	,0.01	1.14	,0.12						
	***P*** ^**c**^	.989		.345		.418							
Dry hot towel vs. Moist hot towel	**MD [95%CI]**	-0.01	[-0.11–0.13]	0.07	[-0.07–0.22]	0.07	[-0.04–0.19]						
	***t*, d**	0.15	,0.01	1.19	,0.12	1.19	,0.05						
	***P*** ^**c**^	.188		.463		.882							

*CI* conference interval, *df* degree of freedom, *MD* mean difference, *SD* standard deviation

^a^A mixed-linear model for two-way repeated-measures ANOVA

^b^One-way repeated-measures ANOVA and Dunnett's test (T1 = control; * P < .01)

^c^One-way repeated-measures ANOVA and Bonferroni correction (*P* < .0167)

There was no significant difference in the ratio of venous assessment scores between using the hot pack and dry hot towel, and approximately 90% of the participants had a score of 3 or higher at T3 (hot pack, 95.5%; dry hot towel, 89.7%). However, the ratio of these scores significantly differed between the moist towel and the other two heat applications at T3 (*P* < .001, Table 4).

**Table 4:** Comparison of changes in venous assessment score (*n* = 88)

**Venous assessment score**		**Hot pack**		**Dry hot towel**		**Moist hot towel**		**Hot pack vs**	**Hot pack vs**	**Dry hot towel vs**
								**Dry hot towel**	**Moist hot towel**	**Moist hot towel**
		**N (%)**						***P***^***a***^		
T1 Baseline	1: neither visible nor palpable	31	(35.2)	35	(39.8)	36	(40.9)	.761	.669	.945
	2: visible but not palpable	9	(10.2)	10	(11.4)	13	(14.8)			
	3: barely visible and palpable	38	(43.2)	31	(35.2)	28	(31.8)			
	4: visible and palpable	10	(11.4)	12	(13.6)	11	(12.5)			
	5: clearly visible and easily palpable	0	(0.0)	0	(0.0)	0	(0.0)			
T3 After tourniquet	1: neither visible nor palpable	3	(3.4)	6	(6.8)	24	(27.3)	.714	** < .001**	** < .001**
	2: visible but not palpable	1	(1.1)	3	(3.4)	8	(9.1)			
	3: barely visible and palpable	54	(61.4)	53	(60.2)	39	(44.3)			
	4: visible and palpable	19	(21.6)	17	(19.3)	15	(17.1)			
	5: clearly visible and easily palpable	11	(12.5)	9	(10.2)	2	(2.3)			
***P***^**b**^		** < .001**		** < .001**		.194				

^a^Freeman–Halton extension of Fisher’s exact test and Bonferroni correction (*P* < .0167); ^b^Freeman–Halton extension of Fisher’s exact test

### Changes in skin temperature and subjective evaluation of the warmth

**Fig. 4:**
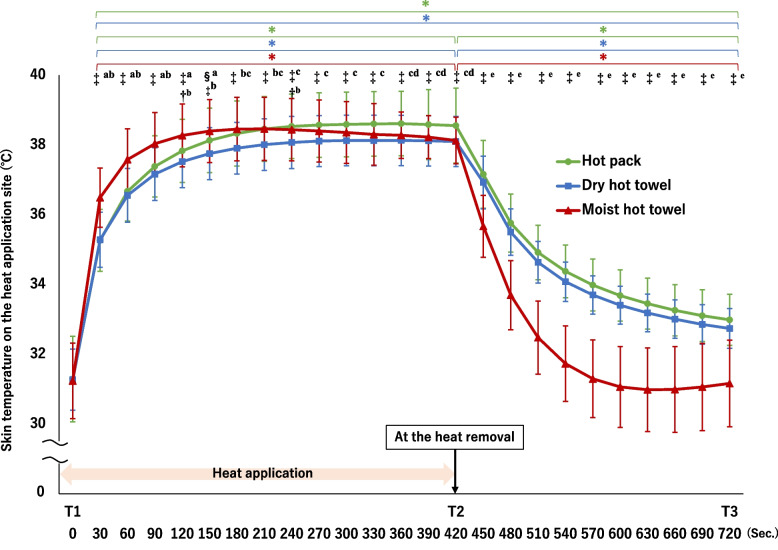
Changes in the skin temperature on the heat application site (*n* = 88). A mixed linear model for two-way repeated-measures ANOVA; One-way repeated-measures ANOVA and Dunnett's test (T1 = control; * P < .001); One-way repeated-measures ANOVA and Bonferroni correction (†*P* < .0167, ‡ *P* < .001, a: moist hot towel > hot pack b: moist hot towel > dry hot towel, c: hot pack > dry hot towel, d: hot pack > moist hot towel, e: hot pack and dry hot pack > moist hot towel). T1, baseline; T2, at the removal of heat; T3, after applying the tourniquet

Skin temperature at the intervention site had significant main effects among heat applications (F [2, 6959.0] = 746.88, *P* < .001), time (F [26, 6959.0] = 3838.08, *P* < .001), and interaction (F [52, 6959.0] = 87.97, *P* < .001). The dry hot towel from 120 s to T2 was significantly lower than that of the hot pack (*P* < . 01, Fig. 4). In contrast, the difference in temperature between the hot pack and two-towel methods at T2 was approximately 0.4 °C (mean [95% CI] °C of difference, dry hot towel:0.45 [0.21–0.69]; moist hot towel:0.43 [0.19–0.67], *P* < .001 for each). There was no significant difference between using the hot pack and the dry hot towel from 450 s to T3, and both at T3 maintained significantly higher temperatures than at T1 (mean [95% CI] °C of difference, hot pack:1.70 [1.50–1.89], dry hot towel:1.47 [1.34–1.59], *P* < .001 for each). On the other hand, the moist hot towel application showed a significantly higher temperature than the other two heat applications at 30–150 s (*P* < .001), but gradually decreased after 210 s. The temperature of the moist hot towel from T2 to T3 was significantly lower than that of the other two heat applications (*P* < .001 for all).

**Fig. 5:**
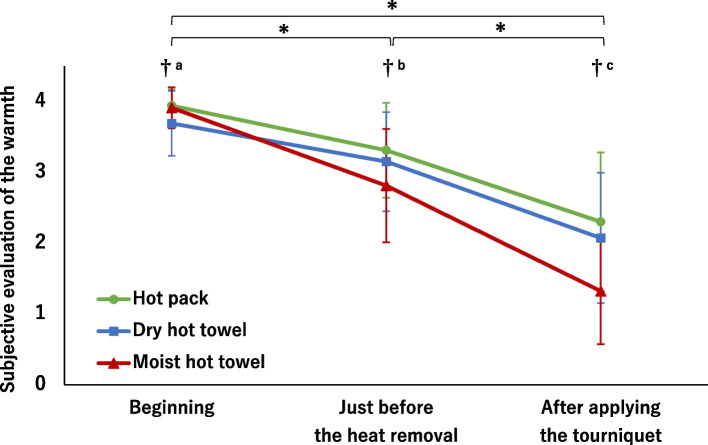
Changes in subjective evaluation of the warmth. Subjective evaluation scores of the warmth: 0 = feeling cold, 1 = feeling cold slightly, 2 = feeling nothing, 3 = feeling a little warm, 4 = feeling warm. A mixed-linear model for two-way repeated-measures ANOVA; One-way repeated-measures ANOVA and Dunnett's test (at the beginning = control; all interventions were * P < .001): One-way repeated-measures ANOVA and Bonferroni correction (†*P* < .001, a: hot pack and moist hot towel > dry hot towel, b and c: hot pack and dry hot towel > moist hot towel)

Subjective evaluation of the warmth also had statistically significant main effects among the heat applications (F [2, 657.7] = 76.04, *P* < .001), time (F [2, 271.5] = 353.42, *P* < .001), and interaction (F [4, 603.9] = 46.28, *P* < .001). The use of the dry hot towel was significantly lower at the beginning than at the other two heat applications (*P* < .001 each, Fig. 5). Thereafter, however, the use of the moist hot towel scored significantly lower than that of the other two heat applications (just before removal: *P* < .001, after tourniquet: *P* < .001).

### Changes in SCH after applying and removing heat

There was no significant difference in the amount of change between the use of the hot pack and the dry hot towel. In contrast, the moist hot towel significantly increased SCH during heating (T2 – T1, *P* < .001) and significantly decreased SCH after heating (T3 – T2, *P* < .001) compared to the hot pack (Table 5).

**Table 5:** Comparison of changes in stratum corneum hydration (*n* = 88)

SCH (A.U.)		**Change in heating = T2 – T1** **(After heating – Baseline)**	**Change after removed = T3 – T2** **(After tourniquet – After heating)**
Hot pack	**Mean (SD)**	12.2 (8.4)	-5.3 (6.4)
Dry hot towel		10.2 (8.3)	-5.1 (6.0)
Moist hot towel		35.1 (18.8)	-28.4 (14.8)
Hot pack vs. Dry hot towel	**MD [95%CI]**	2.00 [-2.48–6.59]	0.19 [-3.45–3.83]
	**t, d**	1.00, 0.24	0.12, 0.05
	***P***^**a**^	.319	.907
Hot pack vs. Moist hot towel	**MD [95%CI]**	22.87 [18.91–26.84]	23.30 [19.69–26.92]
	**t, d**	11.39, 1.42	14.24, 1.81
	***P***^**a**^	< .001	< .001
Dry hot towel vs. Moist hot towel	**MD [95%CI]**	24.88 [20.43–29.33]	23.30 [20.11–26.50]
	**t, d**	12.48, 1.56	14.41, 1.83
	***P***^**a**^	< .001	< .001

*A.U.* arbitrary units, *CI* conference interval, *MD* mean difference, *SCH* stratum corneum hydration, *SD* standard deviation

^a^A mixed-linear model for one-way repeated-measures ANOVA and Bonferroni correction (*P* < .0167)

## Discussion

This study compared the venous dilation effect of using a hot towel (moist and dry heat) with a hot pack before applying the tourniquet for PIVC insertion. Our results showed that the moist hot towel was significantly inferior to the hot pack and the dry hot towel in terms of venous dilation under identical conditions. However, there was no significant difference between the dry hot towel and the hot pack. Therefore, dry hot towels and hot packs are suggested as effective venous dilation strategies for heat application before PIVC insertion.

Although dry hot towels (cotton and water) and hot packs (gel sealed in dry plastic) are made of different materials, the effect sizes of the differences in venous diameter between the two methods were negligible, and approximately 90% of the participants improved to a palpable dilated state after tourniquet use. The skin temperature after tourniquet (in other words five minutes after the heat removal) was not significantly different between these methods and remained significantly higher than that before (mean [95% CI] of difference, hot pack: 1.70 [1.50–1.89] °C, dry hot towel: 1.47 [1.34–1.59] °C). Therefore, both methods could apply heat to the skin temperature that could continuously promote venous dilation for seven minutes. Consistent with previous studies that used a hot pack [9, 11], the hot pack and the dry hot towel significantly increased venous diameters before and after the intervention compared to tourniquet only in this study. Thus, it can be inferred that dry hot towels also effectively promote venous dilation before applying a tourniquet.

On the other hand, there were two reasons why the venous dilatation effect of using the moist hot towel before PIVC insertion was inferior to that of the other two heat applications. First, the heat of the moist hot towel dissipated to the skin and outside air in a short time, resulting in less thermal stimulation of the skin by the moist hot towel at the end of heat application than by the hot pack. This is supported by the results that the moist hot towel had the fastest peak out of skin temperature during heating, and subjective evaluation of the warmth just before removal was also significantly lower than that of the hot pack. The second reason is probably because the heat of vaporization [37] occurring from the use of the moist hot towel was greater than that of the hot pack from removal heating to tourniquet application. The moist hot towel increased the SCH during heat application more than the other two heat applications and evaporated significantly after heating. This was supported by the result that the skin temperature of the moist hot towel after heating was significantly lower than that of the other two heat applications. Our results of the venous diameter and venous assessment score are consistent with the results of Fink et al. (2009) that the success rate of PIVC insertion for moist heat stimulation was inferior to that of dry heat stimulation [12]. In addition, the venous dilation effect resulting from the application of the moist hot towel was inferior to that of the only tourniquet. This result could be attributed to the tendency of the veins affected by the moist hot towel to contract before tourniquet application due to the vaporization heat generated after heating. Therefore, the moist hot towel application would not be recommended.

Our results, which clarified that dry hot towels promote venous dilation as well as hot packs, are a new finding in medical practice where heat application methods are varied. In the future, it could be recommended to use hot packs or dry hot towels to promote venous dilation before PIVC insertion. The dry hot towel in this study was a cotton face towel available in ordinary households that could be easily prepared by simply warming a face towel in hot water, wringing it out, and wrapping it in a plastic bag. Therefore, dry hot towels could be an alternative to hot packs for in-home nursing care and disaster situations where hot packs cannot be prepared. Conversely, the venous dilation effect induced by moist hot towels is not only inferior to that of dry hot towels, attributed to the influence of vaporization heat, but also falls short of the results achieved by employing a tourniquet alone. Thus, when using a hot towel, the pivotal step of enclosing it in a plastic bag is essential. It is necessary to examine the difference in the success rates of PIVC insertion between dry hot towels and hot packs for patients in the future.

This study had some limitations. First, the study population was limited to female students aged 18–29 years. Second, while there was a notable level of agreement in venous assessment score between the researcher and the expert nurse, the evaluator of this study was limited to a sole researcher.

## Conclusions

This study showed that a dry hot towel application for an access site for PIVC insertion promotes venous dilation as well as using a hot pack. Considering the conditions of practice in hospitals and the home environment, a method in which a towel warmed in hot water is wrapped in a dry barrier and applied may be an alternative to a hot pack.

### Supplementary Information

**Additional file 1**.

## Data Availability

The datasets during and/or analyzed in the current study are available from the corresponding author upon reasonable request.

## Abbreviations

ANOVA: Analysis of variance
A.U.: Arbitrary units
BMI: Body mass index
BP: Blood pressure
CI: Confidence intervals
df: Degree of freedom
LSM: Least square mean
MD: Mean difference
PR: Pulse rate
SD: Standard deviation
SCH: Stratum corneum hydration
PIVC: Peripheral intravenous catheter
