# Correction: Venous dilation effect of hot towel (moist and dry heat) versus hot pack for peripheral intravenous catheterization: a quasi-experimental study

**DOI:** 10.1186/s40101-024-00357-4

**Published:** 2024-02-13

**Authors:** Kae Yasuda, Inaho Shishido, Michito Murayama, Sanae Kaga, Rika Yano

**Affiliations:** 1 Graduate School of Health Sciences, Hokkaido University, N12, W5, Kita-Ku, Sapporo, Hokkaido 060-0812 Japan; 2 Faculty of Health Sciences, Hokkaido University, N12, W5, Kita-Ku, Sapporo, Hokkaido 060-0812 Japan

**Correction: J Physiol Anthropol 42, 23 (2023)**

**https://doi.org/10.1186/s40101-023-00340-5**

Following publication of the original article [1], the authors reported errors in Table 4. For the p-values in the comparison of T1 (Baseline) and T3 (After tourniquet) for each condition, the p-values for Dry hot towel and Moist hot towel were switched.

Incorrect: Dry hot towel, *p* = .194; Moist hot towel, *p* < .001

Correct: Dry hot towel, *p* < .001; Moist hot towel, *p* = .194

The original article [1] has been corrected.
